# Temporal Encoding in a Nervous System

**DOI:** 10.1371/journal.pcbi.1002041

**Published:** 2011-05-05

**Authors:** Zane N. Aldworth, Alexander G. Dimitrov, Graham I. Cummins, Tomáš Gedeon, John P. Miller

**Affiliations:** 1 Center for Computational Biology, Montana State University, Bozeman, Montana, United States of America; 2 Department of Mathematical Sciences, Montana State University, Bozeman, Montana, United States of America; 3 Department of Mathematics and Science Programs, Washington State University, Vancouver, Washington, United States of America; Max-Planck Institute of Neurobiology, Germany

## Abstract

We examined the extent to which temporal encoding may be implemented by single neurons in the cercal sensory system of the house cricket *Acheta domesticus*. We found that these neurons exhibit a greater-than-expected coding capacity, due in part to an increased precision in brief patterns of action potentials. We developed linear and non-linear models for decoding the activity of these neurons. We found that the stimuli associated with short-interval patterns of spikes (ISIs of 8 ms or less) could be predicted better by second-order models as compared to linear models. Finally, we characterized the difference between these linear and second-order models in a low-dimensional subspace, and showed that modification of the linear models along only a few dimensions improved their predictive power to parity with the second order models. Together these results show that single neurons are capable of using temporal patterns of spikes as fundamental symbols in their neural code, and that they communicate specific stimulus distributions to subsequent neural structures.

## Author Summary

The information coding schemes used within nervous systems have been the focus of an entire field within neuroscience. An unresolved issue within the general coding problem is the determination of the neural “symbols” with which information is encoded in neural spike trains, analogous to the determination of the nucleotide sequences used to represent proteins in molecular biology. The goal of our study was to determine if pairs of consecutive action potentials contain more or different information about the stimuli that elicit them than would be predicted from an analysis of individual action potentials. We developed linear and non-linear coding models and used likelihood analysis to address this question for sensory interneurons in the cricket cercal sensory system. Our results show that these neurons' spike trains can be decomposed into sequences of two neural symbols: isolated single spikes and short-interval spike doublets. Given the ubiquitous nature of similar neural activity reported in other systems, we suspect that the implementation of such temporal encoding schemes may be widespread across animal phyla. Knowledge of the basic coding units used by single cells will help in building the large-scale neural network models necessary for understanding how nervous systems function.

## Introduction

A considerable amount of research has been focused on determining the information coding schemes used within nervous systems. This is due not only to the intrinsic interest in the nature of the neural code, but to the necessity of understanding the coding scheme implemented within any particular system before a valid model can be developed for the mechanisms underlying neural computation in that system. One important facet of the general coding problem is the determination of the neural symbols with which information is encoded in neural spike trains. Specifically, is all of the information encoded in the mean firing rates of the cells, or is some significant proportion of the information encoded in more complex statistical features of the spike patterns? In the studies reported here, we examined the extent to which temporal encoding is implemented by a set of sensory interneurons in the cercal system of the house cricket, *Acheta domesticus*. To do this, we addressed four related questions: are temporal patterns of spikes reliably elicited by stimuli? Does reliability lead to increased capacity to transmit information? Do temporal patterns represent novel stimulus features? Can any apparent temporal encoding be explained by simple modification to existing models?

Our general approach was to determine if spike patterns elicited in response to sensory stimuli contain more or different information about the stimulus waveform than would be predicted from a simple linear analysis based on a consideration of individual spikes. While a non-linear code could potentially provide more information about the environment to an organism, a simple linear code can be more precisely defined by experimenters, owing to the simplicity of its structure. To that effect, we have utilized the framework of reconstruction analysis pioneered by Bialek and colleagues. In particular, we examined linear stimulus reconstruction, a form of analysis which implicitly assumes the implementation of a linear rate coding scheme [1]–[4], albeit at an arbitrarily fine temporal scale. In order to obtain an estimate of the rate that information about the stimulus is encoded in the neural response (the mutual information rate), the stimulus reconstruction method makes explicit assumptions of what aspects of the stimulus are encoded in the neural response (the reconstruction filter) and how they are encoded by the neural response (by independent single spikes) By contrast, ‘direct’ methodologies [5]–[7] allow exact estimates of the mutual information transmission rates of neurons with few assumptions, but provide no estimates of the stimulus quantities encoded nor the coding scheme implemented by the neurons. Consequently, calculations of mutual information using the direct method can include contributions due to temporal patterns of spikes, as well as the spike rate assumption from the stimulus reconstruction methodology. Comparisons of information rates calculated using the two methods show that linear methods routinely underestimate the true amount of information contained in neural activity [8]. An open question in neural coding is whether this discrepancy arises because neurons use temporal encoding to represent the stimulus space (a possibility explicitly rejected by linear reconstruction), or whether the information gap is caused by other nonlinearities [3].

Previous studies in invertebrate sensory systems, including the cricket cercal system, indicated that linear coding schemes have difficulty describing the stimuli preceding short-interval, high temporal frequency doublets [9]–[12]. We therefore narrowed our investigation to study only short-interval doublets, analogous to the study of bursts in other sensory systems [13]–[16]. Our first step was to determine if stimulus-elicited, short-interval spike doublets occurred with greater precision than would be expected, based on the observed statistics of single spikes. Specifically, we determined if the timing of spikes in short-interval doublets had a higher covariance than would be predicted from an analysis of the jitter in the stimulus-response timing of isolated spikes [17], [18]. We next developed models to examine the extent to which such differences in temporal precision might affect the ability of neurons to transmit information about the sensory environment. We then determined if the stimuli associated with temporal patterns of spikes were significantly different than what was predicted by linear reconstruction. For this analysis, we developed linear and non-linear models for decoding spike doublets, and compared the capabilities of these two types of decoding schemes for representing the stimuli that elicited such patterns of spikes. We demonstrate that short-interval spike doublets convey information at higher rates than predicted by the assumptions of linear coding, and that the stimuli associated with such patterns are better predicted by second-order models than by linear models. This indicates that these neurons employ a temporal encoding scheme [3].

## Results

### Statistics of doublet activity

Our working hypothesis was that sensory systems can use short-interval spike doublets to represent stimulus waveforms that are significantly different than the waveforms that would be predicted by the linear sum of two (offset) copies of the average waveform leading up to a single isolated spike. In order to evaluate this hypothesis we made electrophysiological recordings in giant interneurons receiving input from the cercal system of the house cricket *Acheta domesticus*. This sensory system is common to orthopteran insect species, and is composed of at least 22 bilaterally-symmetric pairs of projecting interneurons that mediate detection of low frequency air currents in the vicinity of the animal's body [19]–[23]. These cells make synaptic connections in the terminal ganglion with approximately 2000 afferent neurons, which themselves innervate the filiform hairs of the cercal appendages. In addition to synapsing with the projecting interneurons, the afferent neurons also synapse with approximately 200 pairs of local spiking and non-spiking interneurons, which make connections with each-other as well as with the projecting interneurons [24]. The axons of the projecting interneurons extend from the terminal ganglion to higher processing and motor centers in the thoracic ganglia and the brain [25],[26]. We performed our experiments in two pairs of these cells, giant interneuron classes 10-2a and 10-3a. These cells have been well-characterized both anatomically [20], [25], [26] and physiologically [4], [21]–[23], [27]–[30], and compose a low-frequency subunit of the projecting interneurons sensitive to air movement from all directions within the horizontal plane. In order to determine the encoding properties of these neurons we recorded intracellularly from single axons (n = 40) while stimulating with both repeating and non-repeating sequences of white noise air currents played at the direction of peak sensitivity for each cell.

**Figure 1:**
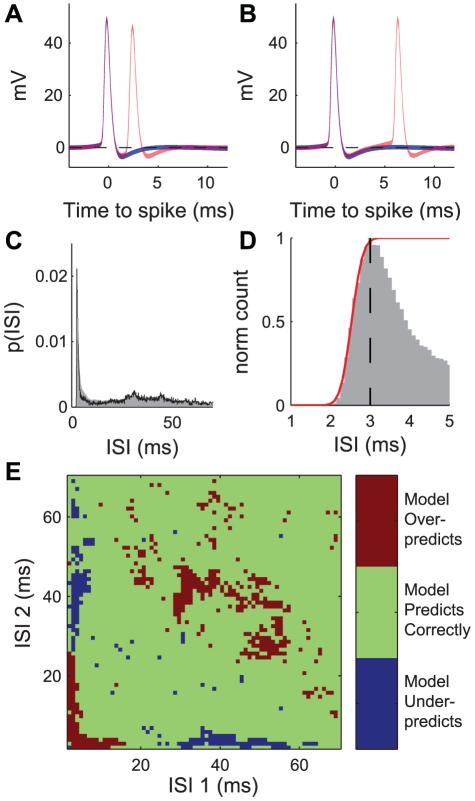
Statistics of doublet spiking. A: ±1 SD envelope showing intracellular voltage waveform relative to resting membrane potential of isolated single spikes (blue) and isolated short doublets of ISI 2.6 ms (red) from a single recording in interneuron of class 10-2a. Dashed black line denotes mean resting membrane potential (0 mV). B: ±1 SD of intracellular waveform from same recording as in A, this time with a doublet of ISI 6.5 ms (red, n = 26). C: ISI histogram of data from recording in A and B at 0.1 ms resolution (black line, n = 26,171 events), as well as compilation data from 40 cells of class 10-2a and 10-3a (gray shaded area, n = 577,435 events). D: Normalized ISI histogram of population data from panel C, with time scale reduced to 1–5 ms. Red line shows the recovery function, with black dashed line showing limits of fit to recovery function. E: Difference between independent model and measurements from data of joint probability of consecutive ISIs. Positive (red) values represent overestimation by the independent model, while negative (blue) values represent underpredictions by the independent model.

In Figure 1 we show the statistics associated with temporal patterns of spikes recorded under these conditions. Panel A shows the mean ± 1 SD of the membrane potential during single spike firing events (blue, n = 10,701 events) as well as during a short doublets of ISI = 2.6 ms (red, n = 464 events) from a single recording of giant interneuron 10-2a. We see that for these short doublet events the second spike occurs while the membrane is still hyperpolarized from the first spike. In contrast, panel B shows the single spike events superimposed with a doublet event with ISI = 6.5 ms (red, n = 26 events) from the same recording. In this case we see that the voltage across the cell membrane has returned to the resting membrane potential (denoted with the broken black line) before the second spike occurs.

Panel C shows the probability of occurrence of all interspike intervals of less than 70 ms (the ISI histogram, binned at 0.1 ms resolution) from the same recording as in panels A and B (black line). In addition, the combined ISI histogram from 40 different cells of class 10-2a and class 10-3a, recorded under the same stimulus conditions, is shown with the gray shade. In the case of the data from the single cell (black line), >85% of all ISIs were of 70 ms or less, while in the data pooled across all cells (gray shade) >90% of the ISIs occurred in this interval. The histogram from the single cell is well within the range of the population data. The ISI histogram contains three clear peaks, one at 44 ms, one at 31 ms, and the tallest peak at 3 ms, which lies just at the edge of the observed hard refractory period for this cell (2 ms). Note that the peaks at 44 and 31 ms correspond to firing rates of 23 and 32 Hz, respectively, which in turn corresponds to the region of peak stimulus-response coherence from analyses associated with stimulus reconstruction [4], [27], [28], [31]. This means that from the perspective of linear rate encoding implicit in stimulus reconstruction, spikes with ISIs in the range of 31–44 ms would carry the most information about the stimulus.

Panel D shows an expanded view of the ISIs from 2–5 ms in the population histogram, with the y axis normalized to 1 at the most often occurring ISI (3 ms). At this time base it becomes clear that the ISIs from the minimum observed (2 ms) to the modal value (3 ms) follow a sigmoidal curve. Berry and Meister [32] showed that the relative refractory period of a neuron can be well described by modeling this sigmoidal curve as a cumulative density function of the ISI probability in this range. In this spirit we fit our data with a Normal CDF (mean = 2.5 ms, SD = 0.2 ms) for later modeling- see Figures 4 and 5.

In order to determine whether or not correlations between spikes could be explained simply by doublet spike patterns, we looked at patterns of two consecutive ISIs (i.e. triplet patterns). If each doublet event was independent of the preceding and following spiking activity, then the joint probability p(ISI_1_ = x,ISI_2_ = y) could be determined by taking the product of the two marginal probabilities, p(ISI_1_ = x)·p(ISI_2_ = y), which we label as the independent joint distribution, p_ind(x,y). We tested this hypothesis for our pooled ISI data by comparing p(x,y) to p_ind(x,y). Regions where the two probability distributions are not significantly different from each other indicate where consecutive ISIs are independent of each other. Figure 1E shows regions where the two models are different at the 95% significance level (after applying the Bonferroni correction for multiple comparisons [33]). The independent model overpredicts the probability in two separate regions lying along the diagonal, the first for consecutive ISIs of approximately 5 ms or less, the second for consecutive ISIs of approximately 30 to 50 ms (red regions). These correspond with the peak regions from the ISI histogram in 1C. The independent model simultaneously underpredicts the probability of a short ISI being either preceded or followed by a silent period of 30–40 ms (blue regions). We note that the relatively enhanced probability of a long silent period preceding short-ISI doublet events could be explained by the presence of a slow voltage-dependent conductance [34]. Voltage dependent Ca conductances are known to exist in these cells [30], [35]. While this observation may help to pinpoint the mechanism for generating these short doublet response patterns, the relatively small probability of these patterns occurring (either as measured in the data, or under the assumptions of independence) makes it unlikely to have a large impact on information transfer in this study of the system (e.g. Figure 4A).

### Measurement of pattern variability

The variability in spike latency of a single spike plays an important role in determining how much information can be encoded in a neuron's activity. However, it is not yet completely clear whether all spikes experience equal variability regardless of prior activity, or whether the immediate spiking history within a cell can affect the variability of subsequent spikes. To address this question, we measured the variability of doublet spiking in our population of cells to repeated presentations of a white noise stimulus. If variability of spike latency were truly independent of spiking history, we would expect average variability of spike timing to be approximately 1.3 ms, as in the case for isolated single spikes (see Figure 5). In addition, we would expect that the variability of ISIs would be even larger, since in that case an ISI would be the sum (more properly the difference) of two independent random variables. In this case, the variance of an ISI would be equal to the sum of the variances of the component spikes' jitter.

**Figure 2:**
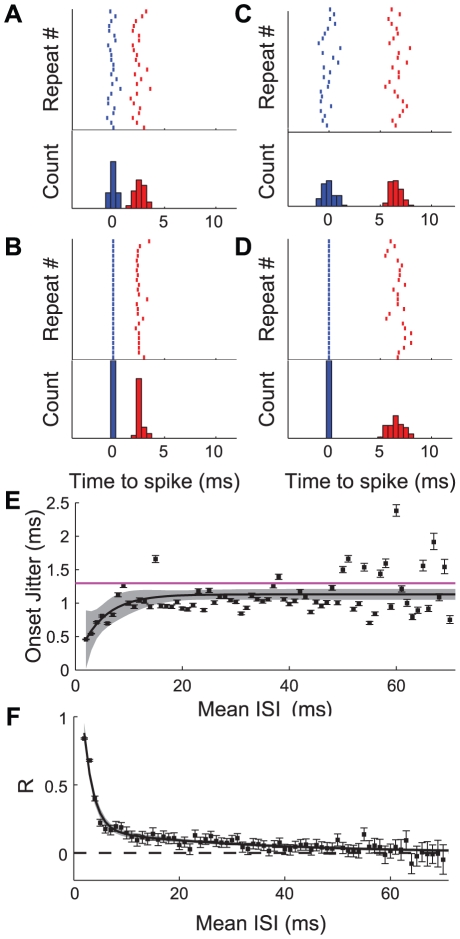
Spike-spike interactions in doublet patterns recorded in cricket interneurons. A, Upper trace: A raster plot showing 25 of 85 responses to repeated presentations of a GWN stimulus, recording from the same cell as shown in Figure 1. The cell consistently responded to the stimulus by firing a doublet (first spike shown in blue, second spike in red) with average ISI of 2.6 ms. A, Lower trace: PSTH of all 85 responses from the raster, with the color convention conserved. B, upper and lower traces: Raster plot and PSTH showing same data from A, here aligned relative to the time of the first spike in the doublet (t = 0) rather than to the timing of the stimulus. This shows the variability in ISI across presentations of a single stimulus. C and D: Data from a second doublet event (mean ISI = 6.5 ms, 73 responses) from the same interneuron, data presentation conserved. E: jitter of arrival time of first spike in repeatable doublets recorded from 40 different cells in 32 animals, as a function of ISI (7753 events composed of 197,601 total pairs of spikes). Black line shows model fit to data (Eq. 1), with shaded area representing 95% confidence envelope around predictions from the model. Horizontal purple line shows population mean of single spike jitter from frozen noise method. F: estimate of correlation coefficient between first and second spikes in repeatable doublets (from same data set as in E). Error bars represent 95% confidence limits on estimation of correlation coefficient. Solid black line shows correlation coefficient as a function of ISI modeled as a double exponential (Eq. 2), with ±95% confidence interval on predictions from the model shown by the shaded grey region.

Figure 2 summarizes the results of the analysis for our 40 neurons. 2A shows 25 of the responses from a single IN 10-2a to 85 presentations of a stimulus that on the average elicited a doublet of 2.6 ms (same cell as in Figure 1A & B). The upper and lower plots show the raster and PSTH of the spiking activity, respectively. The temporal precision of the first and second spikes, as measured by the standard deviation (SD) of the distributions, were 0.3 and 0.5 ms, respectively. Figure 2B shows spiking from the same event, but now conditioned on the first spike of the event rather than the time of the stimulus. The precision of the ISI, as measured by the standard deviation of the difference between the second and first spike times, was <0.3 ms, with a correlation coefficient (R) of 0.8 between the timing of the first and second spikes. Note that the overall ISI response to the stimulus was more precise than the onset latency of either of the individual spikes times. In this case the *a priori* assumption that temporal precision of response is independent of recent spike history can clearly be rejected.

**Figure 3:**
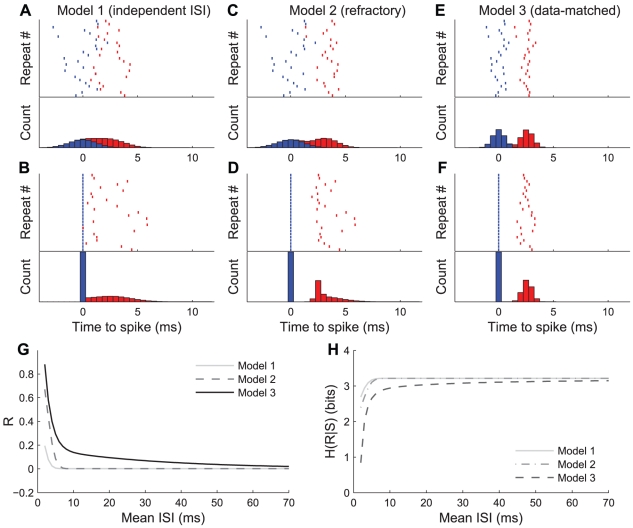
Three models of spike-spike interactions in doublet patterns. A, Upper trace: raster plot of response from cell model 1 (independent ISI) to repeated presentations of a stimulus which reliably elicits a doublet with mean ISI of 2.6 ms, plotting convention as in Figure 2A. Both the first (blue) and second (red) spikes in the doublet are drawn independently from normal distributions with means of 0 and 2.6 ms, respectively, and standard deviations of 1.3 ms. A, Lower trace: Standard PSTH of raster from upper trace, convention conserved from Figure 2. B, Upper and lower traces: raster plot and PSTH showing same data from A with each row aligned to the time of occurrence of the first spike in the response, as in Figure 2B. C and D: (data presentation as in A and B) Model 2 of doublet behavior enforcing a relative refractory period between nearby spikes, using recovery function from Figure 1C and jitter SD of 1.3 ms. E and F: Model 3 (data-matched) of doublet behavior, where the relative timing of spikes is determined by Eqs. 1 and 2. G: Correlation coefficient between timing of first and second spikes of doublets drawn from the three models as a function of ISI. Note that the correlation of Model 3 matches the exponential model from Figure 2F by design. H: Conditional entropy (Eq. 4) of response pattern as a function of mean ISI for all three models.

Figure 2C–D shows raster data and a PSTH for a second event from the same recording as in Figure 3A–B. The mean ISI of this second event was 6.5 ms compared to 2.6 ms in the previous case, while the precision of both spikes within the doublets were similar to the previous case (0.6 ms and 0.5 ms for the first and second spikes of the doublet, respectively). Here however, the distribution of the ISI is slightly larger relative to the two spikes that compose it (precision = 0.7 ms, R = 0.23), although still slightly smaller than expected if the two spikes were independent (0.8 ms, found by taking the square root of the sum of the squared SDs for each spike).

From the data presented in Figure 2 A–D we see that there is clearly a correlation between previous spike history and stimulus-response precision, at least for these two sample firing events in a single cell. We also see that there seems to be a decrease in this correlation with increasing time since the last spike. In order to increase the statistical power of our examination of the temporal precision of ISI events, we pooled the data from 7753 doublet firing events occurring in recordings from all 40 cells in our data set. We first use this larger data set to see if there is systematic variation in the onset precision of a pattern of spikes dependent on the subsequent interspike interval. The results of this analysis are shown in Figure 2E. Here we see that very short doublets are tightly locked to the timing of the stimulus, with a standard deviation across trials (jitter) of less than 0.5 ms for ISIs of 2 and 3 ms. Longer duration ISIs have relatively larger values of jitter, reaching a plateau of >1.1 ms for ISIs of 25 ms or more. The onset jitter as a function of the following ISI was modeled using a simple exponential (Eq. 1, methods) with best-fit coefficients and 95% confidence intervals: x_1_ = −1.0±1.0 ms, x_2_ = 4.8±5.2 ms, x_3_ = 1.1±0.1 ms. The asymptotic value of the onset jitter (x_3_) was similar to the mean stimulus-response jitter of single spikes measured during repeated presentations of frozen noise stimuli (1.3 ms, Figure 5). The resulting model is shown in Figure 2E as the solid black line, with ±95% confidence intervals of the fit shown with the shaded gray regions.

In Figure 2F the same pooled data is used to calculate the correlation between first and second spikes in the doublets as a function of the average ISI of the doublets. What we see in the pooled data confirms what we saw in our earlier example from the single cell. ISIs had correlations significantly different from zero out to approximately 35 ms, and spikes in doublets with short ISIs (<5 ms) have correlations of 0.3 or higher. This means that stimulus events that, on average, elicit short doublet ISIs almost always produced the same response pattern, while stimuli that on average produced ISIs of 10 ms or longer produced sets of doublets with more variable ISIs, as well as the more variable onset demonstrated in Figure 2E. The change in correlation coefficient as a function of ISI was modeled as a double exponential using Eq. 2 (see methods) with the following best fit parameters and 95% confidence intervals: x_1_ = 2.3±0.8, x_2_ = 1.7±0.5 ms, x_3_ = 0.2±0.1, and x_4_ = 28.9±10.7 ms.

### Simulation of distinct stimulus-conditioned spike interactions

In order to determine the potential effects of ISI precision on the ability of a neuron to transmit information, we built three models of doublet firing that differed both in the onset variability of the pattern as well as in the relative timing between spikes in the pattern. Results of the simulations are shown in Figure 3. The first model demonstrates the precision of ISIs if each spike was generated truly independently with variance equivalent to the values measured from response rasters to repeated stimuli (Figure 5). The second model demonstrates the precision of ISIs if each spike was initially generated independently as in the first model, but with a refractory period based on the model of Berry and Meister [32] later enforced in order to move second spikes which occurred within 3 ms of the preceding spike (Figure 1D). Finally, the third model uses onset precision and ISI correlation matched to real data (model curves shown in Figure 2E and 2F, respectively).

Panels 3A–F use the same plotting convention as Figure 2A–D, with each of the three models being displayed in its own vertical column. Although all three models produced variable spike timing raster plots and PSTHs (Figure 3A, 3C, and 3E, upper and lower plots, respectively), the distributions of both spikes relative to the time of the first spike (effectively the ISI- Figure 3B, 3D, and 3F) are distinct between the different models, with correspondingly variable amounts of correlation between first and second spike times.

Figure 3G shows how the correlation coefficient between spike times evolves for each model for average ISIs varied between 2 ms and 65 ms. Note that the correlation for the third model explicitly matches the correlation coefficients calculated from (and are therefore by definition identical to) the data in Figure 2F. The correlations found both in actual data (Figure 2F) as well as in model 3 significantly exceed those for independent and refractory models for ISIs less than 30 ms. The second and third models represent the precision of doublet-spiking according to biophysically plausible mechanisms, while the first model shows doublet spiking as predicted by strict interpretation of the assumptions of linear reconstruction analysis, i.e., independence between spikes. Although the first model has first and second spikes that nominally occur independently of each other, small amounts of correlation are induced by the fact that the earliest spike was always attributed to the first spike distribution, even if it was actually generated from the second spike distribution.

### Information-theoretic analysis of models

In order to rigorously determine the effects the observed precision in ISIs had on a cell's ability to transmit information about the stimuli, we measured information-theoretic quantities such as entropy and mutual information in our data and models. In direct method calculations the mutual information rate is calculated as the difference between two entropies: the total response entropy and the entropy of the response conditioned on a stimulus. The total response entropy determines how much bandwidth a cell has available for representing stimuli, while the conditional entropy reflects how much of that response pattern bandwidth is used to represent the same stimulus. In the present context, a cell could use ISIs with relatively small conditional entropy to transmit more information about a stimulus than ISIs that exhibit relatively large conditional entropies.

Figure 3H shows the contribution to the conditional entropy due to variability in both ISI and timing of pattern onset for each of the three models discussed in the previous section, all as a function of ISI. The conditional entropy curve for model 3 is lower than the curves for the other two models over the entire range tested here, and substantially so for short ISIs. Since model 3 matches data from real cells, while models 1–2 represent decreasingly strict interpretation of linear reconstruction, this indicates that the assumptions of linear reconstruction overestimate the conditional variability of spike patterns. In the information-theoretic framework shown here, this means that a given doublet pattern is capable of transmitting more information about the stimulus than predicted from linear reconstruction assumptions. Specifically, if a cell on average gives a 4 ms doublet response to repeated presentations of a stimulus, model 1 predicts that the conditional entropy of the response would be 3.09 bits, while model 3 predicts that it would only be 2.43 bits. This means that from this specific response event, the relative reduction in the stimulus discrimination ability of model 3 due to noise entropy would be 2∧(3.09-2.43) or approximately two-thirds as large as for model 1.

**Figure 4:**
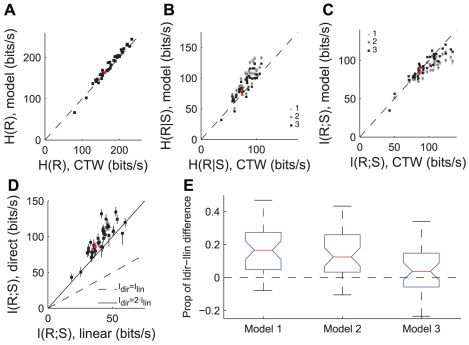
Comparison of information-theoretic quantities. A: Total response entropy rate for 40 neurons as measured using the context-tree-weighting (CTW) technique (x axis), vs. the modeled total response entropy (y axis). In panels A–D the red points indicate values from the cell in Figure 2, dashed black lines indicate unity between the x and y axes. B: Response entropy rate conditioned on a stimulus event as measured by CTW methods (x axis) vs models of the conditional entropy. C: Mutual information about the stimulus contained in the response patterns, calculated as the difference between total and conditional entropies of the response. X axis shows result of CTW estimation for each cell, y axis shows information calculation based on each of the three models. D: Comparison of mutual information measure using linear stimulus reconstruction approach (x axis) with estimation from CTW method. Solid black line indicates I_dir_ = 2·I_lin_ E: Boxplot showing how much of the proportional difference of information between methodologies (I_dir_−I_lin_) can be explained by varying temporal assumptions built in our models. For each of the three models, the boxplot shows the fraction of the information explained by the difference between that model and the direct method estimate from panel D, i.e. prop(x) = (I_dir_−Imodx)/(I_dir_−I_lin_).

In order to determine how much more information could be transmitted overall in neurons using the ISI-correlated precision seen in our cells, we calculated mutual information rates on each of our models using the ‘direct’ methodology [5]. To do this we first calculated the total response entropy using only doublet patterns (i.e., the ISI histogram) for each of our cells. We compared these values to the actual response entropy for each cell, estimated using the context-tree weighting algorithm [7]. The results are shown in Figure 4A. Here each point represents data from a single cell, with the x axis indicating the estimation of the total response entropy from that cell using the CTW method, and the y axis indicating the model estimate of the response entropy described above. In this and all other plots throughout Figure 4, the red symbol represents the various models fit to the exemplar cell shown in Figure 2 A–D. The points all lie along the diagonal, indicating that reducing the response dimensionality to consider ISIs independently does not significantly reduce the calculated value of the response entropy, in spite of the fact that there are correlations in neighboring ISIs (e.g. Figure 1E).

We next fit the free parameters for each of our three models in Figure 3 (jitter values, recovery function, and ISI correlation) to each of our 40 cells. The resulting conditional entropy rates for each of the 3 models and for each of the 40 cells are shown in Figure 4B. Here the x-axis shows the conditional entropy rate estimated using the CTW method. Points that lie along the diagonal match the stimulus-conditioned variability seen in real neurons, while points above or below the diagonal represent overpredictions and underpredictions of conditional variability, respectively. Since model 3 matches the temporal precision parameters from the real data, we expect that it should also be predictive of the conditional entropy of the real cells. We note that this could potentially provide a simple way of estimating information theoretic quantities from relatively few parameters. Figure 4B shows that this is indeed the case- model 3 tends to match the actual conditional entropy calculated in the cell most closely, with results from the other models tending to lie above the diagonal. This means that a strict interpretation of the assumptions of linear reconstruction (model 1) overpredicts the amount of conditional entropy present in the neural activity, and refractory dynamics (model 2) are not sufficient to describe the low variability seen in these neurons.

The information rate for each model was calculated by taking the difference between the total entropy rate and the conditional entropy rate. These values are plotted in Figure 4C vs. the amount of mutual information calculated using the CTW method. As in the case of the conditional entropy, model 3 tended to give the closest match to data, with models 2 and 1 yielding progressively lower estimates of information rate due to their larger relative conditional entropies.

We were concerned with determining how the precise spiking patterns seen in our data affect the ability of these cells to transmit information, and specifically how the assumptions of linear reconstruction might lead to reduced estimates of information rates. Since our models reflect varying degrees of the assumptions implicit in linear reconstruction methods, we compared the modeled information rates with the rates obtained using linear reconstruction for each cell in our sample. Figure 4D shows a comparison between linear reconstruction information rates and rates obtained using the CTW direct method. In almost all cases, the linear method misses more than half of the information available in the spike train. To compare this with the models, we assessed what proportion of the difference between the linear and direct method calculations could be explained by the difference between information from the direct method and our models. The results of this comparison are presented in the boxplot of Figure 4E, which shows the lower and upper quartiles (horizontal blue lines) and the median value (red line) for the proportion of information difference explained across cells. Median values between different models are significantly different at the 95% level if they do not fall within the range of the notch on the respective boxplot. In the data presented here, models 1 and 2 described significant, though statistically indistinguishable proportions of the information difference (16.5% and 12.5%, respectively).

### Quantification of variability in stimulus-response latency

Analysis of the models revealed that correlations imposed by the refractory period only explained a small amount of the discrepancies between direct and linear information estimates. In order to determine if other aspects of precision might explain the information gap, we employed two different methods of assessing the variability of single isolated spikes. These two methods characterize distinct (but related) aspects of spike timing variability. The first method assesses purely biophysical uncertainty by estimating spike onset jitter in the response to ‘frozen’ white noise [18], [36]–[38]. The second method is the ‘dejittering’ technique which assesses temporal uncertainty with non-repeated broadband stimuli [17], [39]–[42]. These two methods use complementary approaches to measure variability. By conditioning on repeated stimuli, the raster method attempts to measure response variability solely due to biophysical sources. In contrast, by using a broader ensemble of non-repeated stimuli, the dejittering technique captures not only biophysical uncertainty, but also latency variance caused by the fact that multiple stimuli are represented by the same response (response invariance). This is an important distinction in the context of comparing linear reconstruction techniques with other measures of information rates in neural systems, since the ‘variability’ in each spike determined by the dejittering method is implicitly included in the construction of the linear kernels.

**Figure 5:**
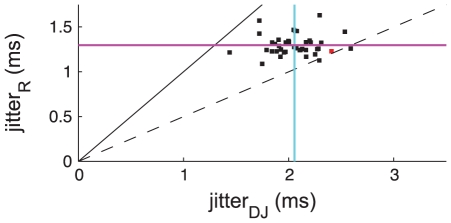
Temporal precision of isolated single spikes. Value along the abscissa shows single spike precision assessed by the dejittering algorithm for 40 cells (population mean shown as a vertical cyan line). Value along the ordinate shows single spike precision assessed by a raster-based analysis for the same cells (population mean shown as horizontal purple line). Each cell is represented by a single point (red point is from same recording shown in Figure 1A–C). The solid black line denotes where the two methods give equal results, while the dashed black line shows where the dejittering method gives a value twice as large as the raster analysis.

Figure 5 shows the results of both analyses on the 40 neurons in our data set. The values along the x axis indicate the standard deviation of the variability in stimulus-spike latency assessed using the dejittering method (mean across the population denoted by the vertical cyan line), while the value on the y axis indicates the standard deviation of the variability in stimulus-spike latency assessed using the raster method (mean across the population is shown with the horizontal purple line). The red point corresponds with the cell indicated in red in Figure 4. As expected from the considerations listed above, the dejittering method consistently gives a larger value for the variability (mean jitter value of 2.1 ms, compared with 1.3 ms for the raster method). We address the fact that there is no significant correlation between the values obtained from the two measures (R = 0.06 across the 40 cells, 95% CI = [−0.26 0.36]) in the following discussion.

To further investigate the relationship between several measured quantities and the difference in information measures (Figure 4D), we used linear regression to determine how well each measurement could predict the information, with the results shown in table 1. The value of the variance obtained using the ‘dejittering’ technique was the only measured value significantly correlated to the information difference, while the precision value from the raster-based method was not significantly correlated. This observation combined with the previously observed lack of correlation between the two variables implies that the discrepancies in information are best explained by accounting for response invariances.

**Table 1:** Linear regression analysis on information rates.

Predictor Variable	Slope	±95% CI	Y Int	±95% CI	R	±95% CI
Jitt_DJ_	0.11	[0.05 0.16]	0.35	[0.24 0.46]	0.54	[0.28 0.73]
Jitt_RB_	0.10	[−0.03 0.23]	0.44	[0.27 0.61]	0.25	[−0.07 0.52]
Firing Rate	0.00	[−0.00 0.00]	0.54	[0.48 0.60]	0.16	[−0.16 0.45]
Burstiness	0.06	[−0.13 0.24]	0.56	[0.51 0.60]	0.10	[−0.22 0.40]
σ_recovery_	−0.00	[−0.00 0.00]	0.57	[0.56 0.59]	−0.01	[−0.32 0.30]

Slope and Y intercept coefficients and their respective 95% CIs from linear regression between five different parameters of models and the proportional difference ((I_D_−I_L_)/I_D_) between direct and linear reconstruction methods of information calculation (from Figure 4D). Also shown is the correlation coefficient R and its 95% confidence intervals. Variables: Jitt_DJ_- temporal precision of isolated single spikes from the dejittering method. Jitt_RB_- temporal precision of isolated single spikes from the raster-based method. Firing Rate- sustained firing rate of the cell during stimulation. Burstiness- proportion of all doublets in recording that have ISIs of 8 ms or less. σ_recovery_- standard deviation of normcdf fit for recovery function from refractory period, from methods of Berry and Meister [32].

This result follows from our previous observation of the lack of correlation between the two variables. The timing variability due to repeated stimulus presentations (the only component of the jitter captured in the raster-based method) affects information calculations using both direct method and stimulus reconstruction approaches. However, the variability in stimulus-response latency for different response events (the component only captured by dejittering) affects stimulus reconstruction-based information estimates, hence the reason it is a good predictor of the gap between the two methods of estimating information.

### Modeling of pattern-conditioned stimuli

**Figure 6:**
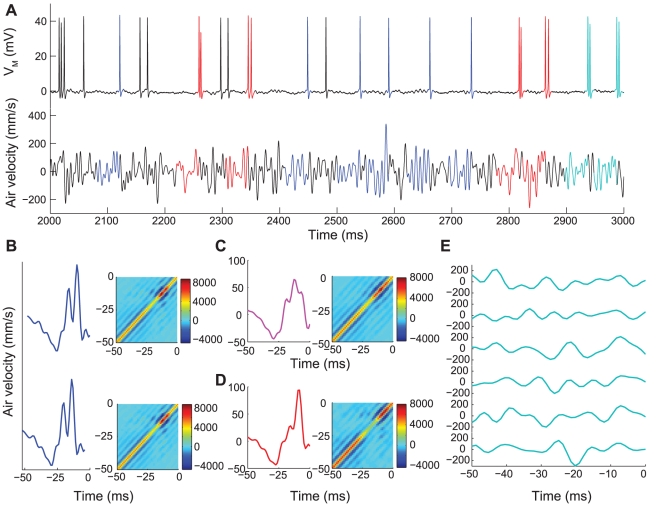
Schematic of modeling event-conditioned stimuli. A: Simultaneous recording of one second of GWN wind stimulus (bottom trace) and intracellular membrane potential (upper trace) from the same interneuron as in Figure 3. Well-isolated response patterns are divided into isolated single spike responses (blue) and ∼2 ms doublets (red and cyan). Response patterns which either are not sufficiently isolated are not considered in subsequent analysis (black). The 50 ms of the stimulus preceding the second spike of the response pattern is highlighted in matching colors (bottom trace). B, Upper panel: Gaussian model of 50 ms of stimulus preceding an isolated single-spike response, consisting of a mean (blue, left panel) and covariance (right panel, color scale in mm^2^/sec^2^) of the entire single-spike-conditioned stimulus ensemble (13,375 events from 30 minutes of recording). B, Lower panel: Same Gaussian model as in upper panel, offset by 2 ms. C: Synthetic Gaussian model of stimulus preceding 2 ms doublets, obtained by summing the means from panel B (cyan, left panel), and summing and then constraining the covariances (Eq. 6). D: Gaussian model (mean, red, and covariance) of 50 ms of stimulus preceding isolated doublet response patterns with 2 ms ISIs, based on 90% of the doublet-conditioned stimulus ensemble (2,652 events from 30 minutes of recording). E: Selection of 6 of the 294 stimulus samples which elicited a 2 ms doublet response and that were not used to build the Gaussian model in panel D, to later be used for likelihood testing.

We wished to determine whether the temporally precise spike doublets represented stimuli that were significantly different from those which preceded single spikes, or from those that would be predicted by linear reconstruction analysis. To do this, we developed a novel likelihood test. First we built models of the stimulus preceding specific patterns of spikes, similar to those developed by de Ruyter van Steveninck and Bialek [9]. For a set of doublets with a specific ISI, the model of the doublet-conditioned stimulus ensemble was generated by taking the mean and covariance across that sample set (schematically depicted for 2 ms ISI in Figure 6A and 6D). This model is referred to as the doublet-triggered stimulus model (DTSM). To build a model of the same ISI that was consistent with the stimulus-reconstruction methodology, we followed the following procedure. First, we collected stimulus segments associated with isolated single spike events, and took the Gaussian approximation of the ensemble as above (Figure 6A and 6B We call this the singlet-triggered stimulus model (STSM). We then took two copies of the same “singlet” model, offset them by the specified ISI, and summed the models (Eqns. 5, 6, 7). This produced a model of the stimuli associated with a doublet that was an extension of the assumptions of linear reconstruction, as discussed in Methods and supplementary text S1. We denote this model as the synthetic doublet-triggered stimulus model (sDTSM, Figure 6C). This procedure provided us with two successively stronger testable hypotheses: 1) the stimuli preceding doublet spiking events were no different from stimuli preceding single spikes, and 2) that the stimuli preceding such doublet patterns could be predicted by an appropriately-combined pattern of the stimuli preceding a single spike. Under this second null hypothesis there are potentially infinite pairs of stimulus-response codewords, limited only by the temporal precision of the stimulus response relationship: a 2.0 ms ISI could represent a different stimulus pattern than a 2.1 ms pattern, etc. In order to properly test these two hypotheses within the constraints of the available data, we examined doublet patterns with at most 1 ms precision. The models were validated by 10× cross-validation (see Methods). Finally, to reduce artifacts associated with the structure of the band-limited stimulus, we projected all models and test data into a reduced-dimensional space (see supplementary text S1). Six of the 294 examples of the test data excluded during the 10× validation for a 2 ms doublet pattern are shown in Figure 6E. Note the variability in individual waveforms relative to the model means shown in 6C and 6D.

### Likelihood analysis

To examine which models could best predict the stimuli preceding doublet events, we performed likelihood ratio tests between the DTSM and STSM, as well as between the DTSM and sDTSM. Considering the log of the ratios, cases in which both tested models were equally likely to explain the data had a log-likelihood ratio value of zero, cases in which the DTSM outperformed the STSM or sDTSM had values >0, while in the reverse case the value was <0.

**Figure 7 pcbi-1002041-g007:**
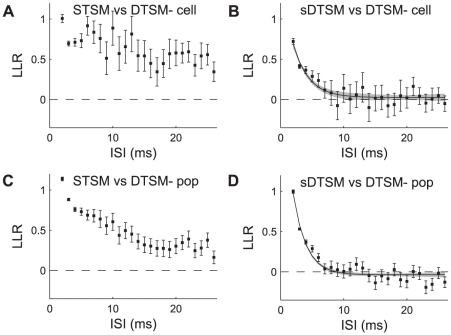
Likelihood analysis. A: Distribution of mean log-likelihood ratios for data-based doublet and singlet models for ISIs ranging from 2–25 ms, from the same cell as in Figure 6. Error bars show ±95% confidence intervals on the mean. B: Distribution of log-likelihood ratios for data-based and synthetic doublet models for same cell as in panel A. Solid black curve shows double exponential model (Eq. 9) fit to data, gray shading indicates 95% confidence interval on predictions from model. C: Distribution of log-likelihood ratios for data-based doublet and singlet models, data pooled across 8 cells, presentation as in A. D: Distribution of log-likelihood ratios for data-based and synthetic models, pooled across 8 cells, as well as exponential model fit to data. Presentation as in panel B.

The results of the likelihood test between the STSM and the DTSM for the same cell as used in Figures 1 and 3 are shown in Figure 7A. For each ISI, the mean ± 95% confidence interval of the log likelihood ratio is shown. The mean estimate of the LLR is greater than zero for all ISIs modeled, which means that, for all ISIs tested in this cell, the stimuli preceding doublets are significantly different from those preceding single spikes.

Data from the same cell were also used to test the sDTSM vs. the DTSM, with the results shown in Figure 7B. The black points show mean ± 95% confidence intervals of the log likelihood ratio as in 7A. The solid line through the distribution shows the least-squares fit of the 4-parametric Eq. 9 to the data (shaded gray region shows ±95% CIs of predictions from the fit). The best fit parameters with 95% confidence intervals were x_1_ = 1.7±0.3, x_2_ = 2.2±0.6 ms, x_3_ = 0.0±0.1, and x_4_ = 2.1×10^5^±3.9×10^10^ ms. Here the second exponential in the mixture is essentially missing, with coefficient close to 0 and uncertain time constant. The higher order model was selected to maintain compatibility with the population case discussed subsequently. In this case, the predicted LLR value from the exponential fit is distinct from zero until ISIs of 8–9 ms (∼4x_2_), indicating that for smaller ISIs the sDTSMs does not account for the data as well as the DTSM.

In order to show which doublets across the set of test cells had log-likelihood ratios indicating a non-linear mapping of stimulus space, we performed the same analysis over the population of cells. To avoid biasing due to small sample sizes and using repeating stimuli, we restricted ourselves to experiments with non-repeating stimuli. This left us with a subset of nine neurons from our initial pool of 40. As before, we estimated LLRs in 10× cross-validation trials for each cell. The results of calculating the LLR for the STSM vs. the DTSM for the nine cells are shown in Figure 7C (plotting convention as in 7A). Here we see again that, as in our exemplar cell, the stimuli preceding single spikes are unable to account for the stimuli preceding doublets.

Finally, we show the population results of the LLRs between the DTSM and the sDTSM in Figure 7D (plotting conventions the same as in 7B). The solid line through the distribution shows the best least-squared fit of Eq. 9 to the data. The best fit parameters with 95% confidence intervals were x_1_ = 2.7±0.2, x_2_ = 2.0±0.2 ms, x_3_ = −0.0±0.1, and x_4_ = −5.9×10^6^±3.1×10^12^ ms. As in the single cell case discussed above, the second term in the double exponential mixture is inactive, while the population LLRs remain significantly positive until ISIs of 7–8 ms.

### Evaluation of compressive non-linearity on models of stimulus

The analysis in the previous section demonstrates that the stimuli preceding patterns of spikes differ significantly from the predictions of linear stimulus reconstruction. However, the specific deviations remain unclear. For instance, one potential explanation for the results seen in Figure 7 is a form of compressive non-linearity, where the stimuli preceding doublets of a specific ISI are better modeled by a sDTSM with a shorter ISI. This would be a natural way for a neuron to adjust its operational range within the limits imposed by biophysical constraints, allowing it to encode stimuli that ‘should’ be represented by an ISI smaller than the cell's refractory period. Such an encoding mechanism would be the representational correlate of the ‘free firing rate’ described by Berry and Meister [32].

**Figure 8 pcbi-1002041-g008:**
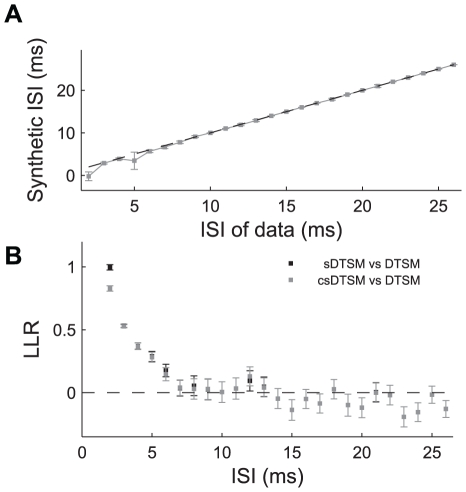
Non-linear compression. A: Non-linear mapping between input ISI (x axis) and best-match synthetic ISI (y axis), determined from peaks in likelihood. B: Effects of non-linear compression on estimates of log-likelihood ratios. Black points show LLR between synthetic (‘synth mod 1’) and data-based doublet models, as in 7D, gray points show LLR between synthetic model modified by non-linear compression (‘synth mod 2’) and data-based doublet models.

In order to determine whether such a mechanism could explain the difference between the sDTSM and the DTSM, we performed a modified likelihood test. Instead of testing whether a data-based or synthetic model best explained observed data with a specific ISI, we asked which of several sDTSMs (each having a different ISI) best explained the data. We built these models using Eqns 6 and 7, for offset values of −3 to 29 ms (in this case a −3 ms offset would be equivalent to a 3 ms offset, but with an additional 3 ms latency prior to the response), and tested them with doublet data containing ISIs from 2 to 26 ms. The results of this analysis are shown in Figure 8, pooled across the 9 cells in our likelihood data set. Figure 8A shows the probability of each sDTSM (y axis, sum along each column = 1) explaining the data for each ISI (x axis), averaged across all cells. For ISIs>2 ms the clear peak lies along the diagonal of the image, indicating that, for these ISIs, the best offset between single spike stimuli in the sDTSM is the actual ISI of the data being modeled. However, for doublets with an ISI of 2 ms, the best-match sDTSM was actually the one with two single spikes at 0 offset (i.e. completely superimposed). This indicates that, on average, doublets of 2 ms and less represent stimuli that are more similar to exactly superimposed copies of stimuli preceding single spikes (rather than offset copies of the singlet-conditioned stimuli). This can be observed by comparing the respective means in Figure 6C and 6D. Such a relationship is consistent with the concept of the compressive non-linearity discussed above.

To see how consistent this relationship was across the cells in our data set, we found the peak probability for each ISI. The mean ± 1 SD of this value across the cells in our population is plotted in Figure 8B, showing that this relationship is indeed consistent across this population of neurons.

Having established that a compressive non-linearity exists in the encoding scheme of these cells, we returned to the likelihood analysis shown in Figure 7D, and repeated it with the best-fit synthetic model, rather than matching the intervals to the ISIs being tested. We refer to this best-fit synthetic model as the compressed synthetic doublet-triggered stimulus model (csDTSM). The results of comparing the DTSM and the csDTSM are shown with grey markers in Figure 8C, superimposed on the original comparison between the DTSM and sDTSM (black markers). Although accounting for the non-linearity significantly increases the predictive power of the 2 ms synthetic model, this improvement still explains only a fraction of the difference in predictive power between the sDTSM and the DTSM for short ISIs. This indicates that the results in Figure 7 cannot be explained solely by the refractory behavior of neurons.

### Quantification of difference in synthetic and data-based models

Having shown that modifications accounting for refractory periods do not explain the differences in our models (Figure 8), we sought to fully quantify these differences in a rigorous manner. To do this we used iSTAC analysis [43] as adapted to multivariate inference in [11]. iSTAC is a form of dimensional reduction, conceptually similar to principal components analysis (PCA). PCA has been used previously to examine the difference between burst- and single spike-triggered stimulus ensembles in model neurons [44]. The difference (and, for this application, the distinct benefit) of iSTAC is that it is guaranteed to preserve the most information about the distinction between the two spaces, as assessed using KL divergence, for any given dimensionality of the subspace (see Methods). The maximally informative subspace of the specified dimensionality provides the most compact description of the difference between the two models [11].

In our case, the two multi-dimensional Gaussian models we wished to compare were the sDTSM and the DTSM. We were interested only in quantifying model differences that were potentially important in decoding responses. Since data-based and synthetic models for ISIs in which the LLR was not significantly different from zero were (by definition) equally good at decoding responses, we focused the comparison on the range for which the data-based model outperformed the synthetic model. For the cell shown in Figure 7B and 8B, this corresponded to ISI<8 ms. Note that although the original dimensionality of these models was equal to the number of sample points in the corresponding event-triggered average (50 points), the comparisons between data-based and synthetic models were performed in the same reduced dimensionality subspace used to calculate the LLRs. iSTAC analysis allowed us to characterize the difference in models using a small number of dimensions, ranging from a single dimension (*i.e.*, a single vector representing the axis of greatest divergence between the two model distributions) up to the full dimensionality of the original models. With iSTAC we could also quantify in bits how much of the difference between the models was captured at each level of reduction.

**Figure 9:**
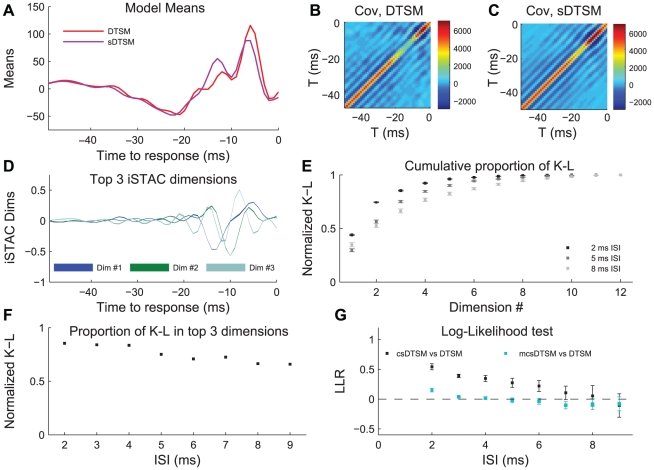
iSTAC analysis of data-based and synthetic models. A: Mean of data-based (red) and synthetic (purple) multivariate Gaussian models for stimulus preceding a 2 ms doublet, from the same cell as in Figures 7A and 7B. Covariance of data-based and synthetic models are shown in panels B and C, respectively (color scale in mm^2^/sec^2^). D: Estimate of the 3 most informative iSTAC dimensions (shaded area indicates mean ± SD across 10× validation). E: Measure of the total normalized K-L divergence between data-based and synthetic models for 2, 5, and 8 ms, as a function of subspace dimensionality. Mean ± SD across 10× validation is shown with error bars, which are on the order of the size of the markers for the points. F: Measure of the portion of the total K-L divergence explained by the subspace containing the three largest iSTAC vectors, as a function of ISI in the model. G: Improvement of the synthetic model performance in LLR tests from the single cell in Figure 7B (black markers) by modification along the 3-dimensional subspace shown in panel 9D (cyan markers). Error bars represent ±95% CIs on the mean.

Figure 9 shows results for iSTAC analysis from the same cell as shown in 7A and 7B. The ISIs shown here were chosen from the region where the doublet outperformed the synthetic model in the LLR test, as represented by the 2 ms ISI models in panels A–D. For the sake of visual clarity, the means, covariances, and iSTAC dimensions in 9A–D are shown in the original 50-dimensional space, even though calculations were performed in the reduced-dimensionality space.

Panel 9A shows the mean of both the DTSM (red) and the csDTSM (purple). These represent the average predictions of the respective models for stimuli preceding a 2 ms response pattern. These two waveforms clearly differ, with the greatest visual difference coming in the regions where the csDTSM overpredicts the stimulus (14 to 12 ms before the response pattern), and underpredicts the stimulus (6 to 3 ms before the response pattern). As shown previously in Figure 7, these differences are significant with respect to coding. The covariance for the DTSM and csDTSM are shown in panels 9B and 9C, respectively. Once again differences in the model are noticeable by eye. Here the diagonal elements from 21 to 9 ms prior to the response are overpredicted by the synthetic model.

The first three vectors describing the maximally informative subspace between the synthetic and doublet models are shown in panel 9. Note that instead of lines expressing the mean of the iSTAC vectors, we use shaded regions to depict the mean ± SD obtained from performing iSTAC on every model from the 10× cross-validation (shaded area = 2SD). In the case of the 2 ms model, the power in the three most informative dimensions is concentrated in a continuous stimulus region from approximately 14 to 3 ms prior to the second spike in the response (Figure 9D). This space corresponds to the regions of dissimilarity from visual inspection, and can be subdivided into a region from 14 to 12 ms before the spike where the synthetic model over-predicts both the mean and variance relative to the data based model, and a second region from 6 to 3 ms prior to the spike where the reverse is true (compare red and purple traces in 9A). This means that, in order to improve the predictive power of the synthetic model the most, we should increase the synthetic mean and covariance in the −3 to −6 ms region, and decrease them in the −12 to −14 ms region. This would be accomplished by scaling along the iSTAC dimensions.

In addition to showing which stimulus dimensions are most informative, we use the iSTAC analysis to quantify the extent to which the DTSM and linear sDTSM differ. This is accomplished by calculating the K-L divergence between the two models for each iSTAC dimension, which gives a measure of how well that dimension explains the difference between the two models in the information-theoretic units of bits (see Methods). The normalized cumulative information recovered for using subspaces of various sizes up to 12 dimensions is shown in Figure 9E for models of 2, 5 and 8 ms ISIs (cumulative K-L divergence without normalization is shown in Figure S1). We see that dimensional reduction with the least loss of information is accomplished with the 2 ms models. In comparison, the longer ISIs require more dimensions to describe an equivalent amount of information about the differences between models. This point is underscored in panel 9F, where we show the proportion of total information contained in the subspace containing iSTAC dimensions 1–3 for each ISI from 2 to 9 ms. Over 85% of the difference is captured by a 3-dimensional subspace for the 2 ms ISI, while only ∼65% of the difference is captured in the case of the 9 ms ISI.

Taken together, these results indicate that for short ISIs, changes in a relatively small subspace of the synthetic model would cause substantial improvements in that model's LLR performance. We tested this notion by modifying the mean and covariance of the synthetic model for each ISI so that they were identical to the mean and covariance of the corresponding DTSMs in a three-dimensional iSTAC subspace, but were unchanged along the remaining dimensions. We refer to such synthetic models as the modified compressed synthetic doublet-triggered stimulus model (mcsDTSM). Figure 9G shows the results of LLR analysis performed for the csDTSM vs. the DTSM, as well as for the mcsDTSM vs. the DTSM, for ISIs between 2 and 9 ms. For each ISI from 2–7 ms, the LLR decreased significantly for the mcsDTSM in comparison with the csDTSM. In this case the LLR stopped being significant at the 95% confidence level for all ISIs greater than 4 ms. Similar results were seen for other cells in the data set (Figure S2). This indicates that the iSTAC dimensions do indeed capture the differences between the models that are important for decoding neural activity. Note that although the 3-dimensional subspace explains the greatest *percentage* of difference for the shortest ISIs (panel 9F), these same ISIs have the greatest LLR difference between sDTSM and DTSM, and hence for the shortest ISIs the mcsDTSM does not quite explain the data as well as the DTSM. These results further indicate that the deviations of these cells from linearity, previously shown in Figure 7, can be quantified using a dimensionally-compact descriptor. These results also help pave the way for future lines of research into the nature of non-linear encoding, including experiments to determine the precise biophysical mechanisms which might lead to the observed deviations from linearity, as well as confirmation of these results by showing whether or not modifications of the stimulus along these few dimensions affect the probability of eliciting short doublets.

## Discussion

### Temporal encoding hypotheses

The nature of the neural code has long been studied. While early work such as that of Adrian showed that much of the information about a stimulus is contained in the firing rate of a neural response [45], more sophisticated analyses have demonstrated that information about the stimulus can be extracted from the timing of individual spikes in the neural response [1], [2], [9]. Additionally, it has been shown that neurons are capable of responding with as much temporal precision as 1 ms [18], [36]–[38], [46]. This has led to the hypothesis that neurons might use a temporal code, through which multi-spike patterns are used to represent stimuli that are distinct from those stimuli which could be predicted based on consideration of individual spikes [3]. Recent work in several systems have purported to show various types of temporal encoding with respect to this definition [47]–[52]. Our results are consistent with this temporal encoding hypothesis, where high frequency doublets (2–8 ms) are used to represent stimuli composed of frequencies less than 200 Hz. The results also indicate how the stimuli corresponding to these doublets differ from those stimuli that can be represented by sums of appropriately offset linear kernels.

### Temporal precision of multi-spike dode words

Several factors have been identified that would act to constrain the effectiveness of temporal codes. In particular, the upper bound on the duration of multiple-spike code words is imposed by the biophysical constraints on decoding and by selective pressure on the reaction time of the animal in making a decision based on sensory input. Similarly, the lower bound on the duration of multiple-spike code words is imposed by the refractory period of the cell and by the limiting temporal uncertainty in the stimulus-response relationship [9], [32], [50]. One specific factor contributing to the temporal uncertainty in stimulus-response relations is the inherent limiting noisiness or “jitter” in spike timing. While cells driven by dynamic, large-amplitude stimuli tend to minimize this jitter [5], [18], [37], [38], [53]–[57], the temporal variability of single spikes must still limit the ability of a neuron to transfer information with precise patterns. This limit would become especially severe if noise from single spikes summed independently. We have recently shown that isolated single spikes recorded from cercal interneurons exhibit a stimulus-to-response jitter of ∼2.2 ms around the mean latency [17]. Here we have repeated that analysis, and further extended it in several manners. First we show that doublet events with ISIs of less than 30 ms have event onset jitter considerably tighter than the onset variability of single spikes across trials (Figure 2E), which is in agreement with modeling studies [44]. Second we show that this stimulus-response jitter does not independently affect spikes in short-interval doublets, and that the times of occurrence of spikes in doublets with average ISIs of 5 ms or less are tightly correlated, exceeding even the precision expected from consideration of a refractory period as shown by Berry and Meister [32]. Such correlations, where repeated stimuli elicit nearly identical patterns of spikes, are necessary for a temporal code to be able to efficiently transmit information about the stimulus. Such mechanisms have been theoretically implicated in models of visual cortex [44], and indeed highly reproducible ISIs have been shown to exist in the presence of noise in several vertebrate sensory systems [32], [50], [58], suggesting that this form of temporal encoding is not restricted to the insect realm.

The analysis reported here also derives an estimate of the minimum ‘word length’ of temporal patterns distinct from single spikes in this set of neurons (≤8 ms), and should be used as a first step in determining parameters for analyses of dynamical neural coding [10], [11], [59].

### Implications for information transmission

Information theoretic analysis has proven to be a useful tool in determining the coding schemes of many different sensory systems. Two of the most popular methods of information theoretic analysis in neuroscience, the direct method and linear stimulus reconstruction, each have distinct advantages. Assuming that biases are appropriately accounted for [6], [7], the former method gives an accurate estimate of the true information rate contained in neural activity and allows for encoding of stimulus parameters by temporal patterns of responses (as well as all other types of responses), however it gives no model for how this transmission occurs. Stimulus reconstruction offers a model for how stimulus energy is encoded by neurons, but only gives a lower bound estimate for information transmission and makes strong assumptions such as precluding the possibility of temporal encoding. Several studies have now performed both analyses on the same data [7], [53], [60], [61], while in other cases different studies have used the two methods separately on similar cells using similar stimuli [1], [5], [16], [52], [62], [63]. These experiments have been performed in different sensory modalities from diverse animal phyla, including retina in salamander, guinea pig, and cat, cat thalamus, primate visual area MT, the fish electrosensory lateral line lobe (ELL), and the fly visual system. In all cases reported so far the linear reconstruction technique has substantially underestimated the information available in the neural activity, in some cases missing 80% of the information (Table 2).

**Table 2:** Comparison of linear and direct estimates of information rates in various sensory systems.

Preparation	Inf Method	Inf Rate	Inf Ratio	Reference
Fly H1	Rev Recon	64	2.5*	[1]
	Direct	81		[5]
Salamander Retina	Rev Recon	3.2	∼3.0	[63]
	Direct	∼9.6		[62]
Guinea Pig Retina	Rev Recon	3.3	4.6	[7]
	Direct	15.2		
Cat Retina	Rev Recon	61.1/62.2	1.4/1.8†	[61]
	Direct	82.5/109.2		
Cat Thalamus	Rev Recon	∼1	∼3.6	[16]
	Direct	3.6		[52]
Macaque MT	Rev Recon	5	2.5	[53]
	Direct	12.5		
Fish ELL	Rev Recon	14.7/25.2	1.6/2.1‡	[60]
	Direct	23.1/52.9		
Cricket Cercal INs	Rev Recon	41.1±7.8	2.3	Present Study
	Direct	96.7±19.8		

Comparison of methods of estimating information rate which either take into account temporal patterns of spikes (direct methods) or which assume independence of consecutive spikes (reverse reconstruction methodologies). All values for information rates are reported as bits/second, except for the values for cat thalamus, which are reported in units of bits/spike.

*The estimate of information rate from linear reconstruction for H1 was actually based on an artificial left/right pair, while the direct method estimate was for a single neuron. The ratio reported here of direct estimate/linear reconstruction estimate was based on one-half of the value from linear reconstruction, as estimates from such artificial pairs tend to double the information estimate of single cells [4].

**†:** Cat Retinal cells were split into four physiological categories- on and off X cells, and on and off Y cells. In this table the four categories were summarized by two numbers, with on and off X cells lumped into one category (numbers on the left for information rates and ratios), and on and off Y cells placed in a second category (numbers on the right).

**‡:** The electric fish ELL was stimulated with two different, behaviorally relevant stimulus geometries: local geometry corresponding to prey signals (numbers on the left for information rates and ratios), and global geometry corresponding to conspecific signaling (numbers on the right).

**§:** Data reported are from same cells as used in the present study. Values reported are mean ± SD, n = 40.

One of the possible reasons for this discrepancy in information rates is that any aspects of the stimuli encoded by temporal patterns in the nervous system would not be accounted for in calculations using stimulus reconstruction (in addition to other proposed non-linearities [8], [53], [61]). Our analysis shows that a significant proportion of this information gap can be attributed to assumptions about the temporal variability implicit in reconstruction methodology (e.g. Figures 3–4 and table 1). This result is in agreement with the work of Bialek and colleagues, as well as several other studies which have measured the information contained in specific patterns of spikes [9], [47], [64]–[66].

In particular, de Ruyter van Steveninck and Bialek showed that in the H1 neurons of flies, ISIs of 10 ms and less transmitted the most information about the stimulus (their Figure 7). Similarly, in the same system Brenner *et al* showed that ISIs of 6 ms and less provided greater “event information” than longer ISIs (their Figure 3). In both studies the authors attempted to estimate the mutual information tied to specific response events. Here we use a complementary approach, instead characterizing only the conditional entropy for specific response events (Figures 3H and 4B), and then relating that to estimates of the total mutual information (Figure 4D–E). Our results are consistent with the work from flies, showing that ISIs less than 10 ms are capable of carrying more information about the stimulus than longer-interval doublets.

Other recent work has shown that the lower bound estimate provided by linear reconstruction techniques becomes looser in the case of high-intensity stimulation [12], [60], [61], [67]. The amplitude of our stimulus was larger than reported values in other investigations of this system [4], [27], [28]. This indicates that part of our measured information gap might be due to our stimulation regime, though direct comparison of stimulation amplitude is confounded by differing calibration methods between the previous studies and the current one. Recent work by Pillow and colleagues [68] demonstrates an alternate method to the lower bound information rates, based on estimation of the *maximum a posteriori* distribution of stimuli conditioned upon responses. This method provides a tighter lower bound on the information rate under high-intensity stimulation conditions, provided there is enough data for estimation of the covariance of residuals to have converged and that the neurons are sufficiently linear. It is possible that using this linear estimator would lead to smaller information gaps in our system as well as the others listed in Table 2, though given the magnitude of the reported gaps (∼60%) it is likely that there would still be a significant discrepancy between linear and direct information estimates.

Previous reports that studied the cricket cercal system provided evidence that interneurons 10-2a and 10-3a as well as their presynaptic afferents strictly use linear encoding [4], [27], [28], [69]. Data that supported these conclusions included consideration of the residual from stimulus reconstruction. However, the potential for patterns of spikes to non-linearly represent stimulus waveforms has not previously been investigated in this system. Our results here show that such patterns of spikes represent distinct stimuli, and are capable of transmitting information at higher rates than can be recovered from spike rate alone. This represents a substantial revision in our understanding of how the cercal system operates.

### Bursting vs. tonic spiking

Although no bursting mechanism has been characterized in the cricket cercal system, we note that the relatively enhanced probability of a long silent period preceding short-ISI doublet events (Figure 1E) is reminiscent of the voltage-dependent calcium conductance (I_T_) involved in the generation of bursting activity in relay cells of the mammalian LGN [34]. Indeed, it is known that there are voltage-dependent calcium conductances in these cells [30], [35], and the deviation from independence of neighboring ISIs observed here is consistent with a calcium conductance-based bursting mechanism that has a time-dependent inactivation mechanism.

Our neural coding results are also in broad agreement with work on bursting activity in the pyramidal cells of the electrosensory lateral line lobe (ELL) of weakly electric fish, the lateral geniculate nucleus (LGN) in the mammalian visual pathway and other sensory systems. In these systems it has been suggested that isolated single spikes and short ISI ‘burst’ events compose two separate channels for encoding information about the stimulus [13], [70]. It has been shown that the stimuli preceding bursts are distinct from the stimuli preceding single spikes [16], [71], and that certain types of naturalistic stimuli are more likely to elicit burst responses [72], [73]. It has also been shown that bursts with distinct ISIs can be clustered into classes representing distinct stimuli [10], [15], [50], [74], [75], and that stimuli associated with bursts are easier to decode using a feature extraction vs. a reconstruction technique [13], [73], [76]. These results have been interpreted as suggesting that tonic spiking in sensory systems is used to keep a ‘running commentary’ of the dynamics of the stimulus, while burst events are used for feature detection of surprising or otherwise ethologically-relevant stimulus events.

The results that we present here agree with this suggestion of segregated tasks. We show that in the case of cercal interneurons single spikes and doublets code for significantly different stimuli. Additionally, we show that spikes belonging to ISIs of greater than 8 ms correspond with stimuli that essentially match appropriately offset copies of the linear reconstruction kernels, and can be thought of as essentially tonic in nature. However, the stimuli associated with the shorter doublets are somewhat larger and sharper than linear predictions (e.g. Figure 6C and D, Figure 9A), and can be thought of as belonging to a separate ‘bursty’ information channel. We also extend previous results by demonstrating explicitly that this bursty channel is not only distinct from tonic spiking as in [16], [71], but also that the associated stimuli are distinct from combinations of the stimuli associated with single spikes (e.g. Figures 6–9). This is in general agreement with results from the H1 neurons of flies as well as auditory receptor neurons in locusts [9], [65], [77]. In the study of de Ruyter van Steveninck and Bialek it was shown that the mean stimulus preceding short patterns of spikes in the H1 neurons of flies was impossible to predict from combinations of the mean stimulus preceding single spikes (their Figures 5G and 12). Similarly, the recent study of Fernandes *et al* (also using H1 neurons) showed that spike-spike interactions on short time scales were significant in determining the shape of second-order reconstruction kernels, and that accounting for them could lead to 100% improvement in reconstruction at specific moments during stimulation. A similar study by Eyherabide and colleagues on grasshopper auditory neurons used T-tests to show that the stimuli preceding bursts could not be predicted by offset copies of the stimulus preceding single spikes. We extend these studies by not only showing that the stimulus preceding bursts comprises a unique codeword in our system, but also showing how these differences can not be related to simple refractory phenomena (Figure 8), as well as the specific dimensions along which linear models fail to predict the stimulus preceding bursts (Figure 9).

### Biological relevance

It is important to consider possible neural coding schemes within a broader neuroethological context. The cercal system of crickets has been shown to be responsive to acceleration due to gravity [78], to the touch of approaching predators [79], [80], to air movement caused by the approach of predators [81]–[84], and to air movement generated by the stridulation of nearby conspecifics [22], [85], [86]. All of these types of stimuli activate the cercal filiform mechanosensors, which synapse onto the interneurons studied here. (The first two types of stimuli also activate several other types of mechanosensory receptors in addition to the filiform hairs, which do not synapse directly onto the interneurons we studied.) Although the precise synaptic-connectivity with higher order neurons is unknown in the cricket cercal system, it is known that these giant interneurons have axonal arborizations in the thoracic ganglia that connect to motor nerves, as well as arborizations in the mechanosensory centers in the protocerebrum [25], [26], and that neurons with multi-modal sensitivities (including sensitivity to air flow) project out of these areas and can effect behavior related to locomotion [87]–[89]. It is unclear what role the cercal system plays in specific behaviors such as phonotaxis and courtship [90], although at the very least the relatively few cercal filiform interneurons must carry enough information to allow the animal to distinguish between the signature of an approaching predator and the infrasound components of conspecific calling songs. A myriad of different encoding schemes for representing this information can be imagined, including one where different post-synaptic neurons use short term depression and facilitation to selectively filter for specific ISI durations in bursts [91]. There is, in fact, strong evidence that crickets specifically use short bursts at the interneuron level of the auditory system to trigger evasive responses [14]. In addition, there is evidence in other orthopteran species that thoracic motorneurons which receive input from the cercal system undergo facilitiation, as short presynaptic bursts trigger spiking response in the motorneurons, while presynaptic single spikes do not [92]. Further, it has been shown that direct intracellular current injection into cricket neurons 10-2a and 10-3a can elicit escape-like running responses within tens of milliseconds [25]. Here we show that stimuli which include the frequency content of both predatory and conspecific stimuli elicit both single spike and doublet spiking responses, and that these two response types represent distinct information about the stimuli. A plausible working hypothesis is that the short-interval spike doublets we characterize here are the symbolic correlate of a component of the animal's evasive response, consistent with the searchlight hypothesis of bursting in other sensory systems [70], while single isolated spikes mediate detection of other sensory signals.

## Methods

### Preparation, electrophysiology, and stimulation

Experiments were conducted on 32 female crickets of the species *Acheta domesticus* that had undergone their final adult molt within the last 8–24 hours. Crickets were anaesthetized by placing them on ice for 5–10 minutes, and then removing the legs, ovipositor, wings, gut, reproductive organs, and fatty tissue. The preparation was pinned to a disk of silicone elastomer, and all incisions were sealed with petroleum jelly. The abdominal cavity was connected to a perfusion system containing hypotonic cricket saline [93], and a small steel platform was inserted under the terminal abdominal ganglion.

Intracellular recordings were made from neurons 10-2a (n = 19) and 10-3a (n = 21), two bilaterally-symmetric pairs of giant projecting interneurons in the terminal abdominal ganglion of the cricket [20]. Sharp intracellular electrode penetration into the axons of these neurons was facilitated by first applying protease solution (Sigma-Aldritch, P5147, St Louis, MO). Electrodes were filled with a mixture of 2% Neurobiotin (Vector Laboratories, SP1120) and 3 M KCl, yielding electrode resistances between 2 and 10 MΩ. During neural recordings the Neurobiotin passively entered the neuron, and following the experiment the neuron was conjugated with an ABC-DAB reaction (DAKOCytomation K0377, and Vector Laboratories SK4100, respectively) for morphological identification.

Experiments were performed in a previously described stimulation system [10], in which air particle displacement generated by stereo speakers stimulated the filiform hairs on the crickets' cerci. Each filiform hair is innervated by an afferent neuron that makes direct excitatory synaptic contact with the giant projecting interneurons. All stimuli consisted of single-dimensional, 10–200 Hz band-passed (BP) Gaussian White Noise (GWN) air movement with an RMS amplitude of 72–76 mm/sec. The amplitude of the air movement was calibrated using a low-velocity air current sensor (Titan sensor, MicroFlown Technologies, Zevenaar, The Netherlands). This band encompassed the range of all known stimuli of ethological relevance to this system [83], [86], [94]. Stimuli were either long-term, non-repeating stimuli of up to approximately 33 minutes length for the stimulus codeword analysis, or else 30 to 100 repeats of a short, 10 second segment for the analysis of temporal variability of doublet spiking patterns. During experiments the membrane potential and stimulus voltage were sampled at 10 kHz and recorded on a Windows XP computer running proprietary LabVIEW software. Prior to analysis all stimulus voltage waveforms were run through a calibration filter to convert them to measures of air particle velocity.

### Measurements of temporal uncertainty

Doublet events that were consistently elicited by repeated presentations of the stimulus were identified with a modified version of the event identification protocol of Berry and colleagues [18]. In order to avoid results due to adaptation, we excluded initial repetitions of the stimulus where the average firing rate was greater than 120% of the average firing rate across all trials. The adapted responses to repeated trials of the stimulus were then binned into histograms at 1 ms resolution and thresheld in order to define firing boundaries of events. Doublets were extracted from the collections of all events, taking care that no more than 20% of the trials contained contaminating spikes. Varying this exclusion threshold between 10–90% of the trials did not greatly affect the results of the correlation analysis. For each doublet event, the timing of the first and second spikes of the doublet on each trial was extracted and pooled across all events and all cells by ISI. The jitter (standard deviation of the first spike time across trials) and the correlation coefficients between first and second spike in the doublet were then calculated. Simple exponential models of the form:
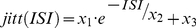
(1)and
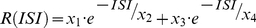
(2)were then fit to the jitter and correlation data, respectively, where *ISI* represents the mean inter-spike-interval and *x_1_* through *x_4_* represent the parameters fit in the optimization. For all exponential equations, the number of parameters used to fit the data was determined by selecting the model with the lowest value of the Akaike Information Criterion (AIC) [95]. Fits for the coefficients, 95% confidence intervals on the coefficients, and 95% confidence intervals on predictions from the models were obtained with least-squares fitting using the routines *nlinfit*, *nlparci* and *nlpredci* from the MATLAB® statistics toolbox.

### Modeling of ISI timing precision

Three models of ISI variability were constructed in order to elucidate the mechanisms of ISI precision seen in real cells. For the first two models the onset jitter (defined as the standard deviation in first spike timing across repeated presentations of identical stimulus waveforms) was fixed at 1.3 ms, which is the observed across-trial jitter of isolated single spikes in recordings from the 40 recorded cells (Figure 5). In model 1 the timing of the two spikes in each trial were drawn from two independent normal distributions with SD of 1.3 ms. In model 2, spike times were drawn independently from normal distributions as in model 1, however second spikes that occurred within a refractory period (Figure 1) were moved by a Gaussian random variable with SD determined from the recovery function fit to the ISI histogram (Figure 1D) [32]. This approximated the presence of a refractory period. For the third model, both onset jitter of the doublet and variability within the doublet were matched to values observed from data (equations 1 and 2). Correlations between the spikes were imposed by multiplying the time of the first spike (mean time of occurrence = 0) by *R* and adding a sample from a normally-distributed random variable with variance
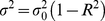
(3)where *σ*_0_ is the standard deviation of the first spike. This preserved the unconditioned variance of the second spike time, but constrained the variance of the ISI between the first and second spike (the covariance) to be less than the sum of the variances of the two separate distributions.

### Information-theoretic calculations

Models of ISI variability and onset precision were used to calculate information rates relating to various assumptions on the correlation between nearby spikes. This was done by assuming independence between onset jitter and ISI variability. For each ISI in each model, the probability of a response pattern conditioned on a stimulus was approximated by determining the temporal correlation between spikes for the ISI/model pair, determining the variance of the corresponding ISI using equation 3 (representing the variability of ISIs conditioned on a stimulus), and then adding the onset jitter squared appropriate to each model (representing the variance in latency of patterns conditioned on a stimulus, see previous section). The square root of the resulting sum was used as the standard deviation to generate a normal probability density function representing the total pattern variability conditioned on the occurrence of a stimulus. The conditional entropy for the ISI, *H_C_*, was calculated according to
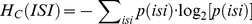
(4)


This yielded the conditional entropy per stimulus event. To transform this into a rate we weighted by the probability of each ISI occurring in our data set (we used the ISI histogram as a surrogate, representing the probability of our white noise stimulus eliciting a given pattern), and then multiplied this value by the rate of occurrence of ISIs in the recording (number of spikes in the recording-1 divided by the length of the recording). The unconditional or total response entropy rate was calculated using only the ISI histogram plugged into Eq. 4, multiplied by the rate of occurrence of ISIs. The mutual information of the models was estimated as the difference in the two entropy rates. These model values were compared with entropy and information rates calculated from our data using the CTW method [7], as well as information rates using stimulus reconstruction methods [1], [2] obtained through a multi-taper calculation of the coherence function [4], [96].

### Response-conditioned stimulus models

Three distinct response-conditioned stimulus models were developed: two doublet-conditioned models and a singlet-conditioned model. For the first doublet-conditioned model, all of the well-isolated doublets with a given inter-spike-interval, which were neither preceded nor followed by other spikes within a 20 ms window, were located. Note that this definition of doublets necessarily differs from the definition used in finding doublet responses from repeated presentations of a single stimulus described in the previous section. The stimulus segments starting 50 ms prior to the second spike of all doublet events were collected to form the doublet-triggered stimulus ensemble (DTSE). 10% of these stimuli were held out for later cross-validation as the test doublet-triggered stimulus ensemble (tDTSE), while the remaining 90% of the ensemble was used to build the doublet-triggered stimulus model (DTSM). This consisted of the mean, *μ_D_*, and the covariance matrix of the ensemble, *C_D_*, both sampled at 1 kHz.

In order to build the singlet model and the second doublet model, we identified all of the single spikes isolated by the same criteria used for the doublets (i.e., no other spikes in a 20 ms window around the spike). All of the stimulus segments preceding the isolated spikes were collected, extending from 50 to 1 ms prior to the spike. The entire singlet-triggered stimulus ensemble (STSE) was then used to build a singlet-triggered stimulus model (STSM) with mean *μ_s_* and covariance *C_s_*. In order to form a synthetic mean of the stimulus for the doublet (*μ_sD_*), *μ_s_* was replicated, shifted in time, and summed according to:
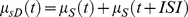
(5)where *ISI* represents the inter-spike interval of the desired model. The synthetic covariance of the stimulus for the doublet (*C_sD_*) was calculated according to:
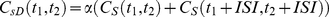
(6)where
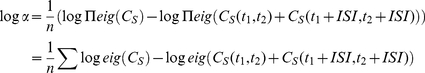
(7)ensured that the sDTSM operated over approximately the same volume in stimulus space as the DTSM. Products and sums in (7) are over all eigenvalues of the respective covariance matrices. See the supplementary text S1, section ‘Covariance Structure of Synthetic Stimulus Models’ for derivations of Eq. 6 and Eq. 7. *μ_sD_*, and *C_sD_* composed the sDTSM.

### Likelihood tests

The relative abilities of the STSM, the sDTSM, and the DTSM to predict the stimuli preceding a doublet response were tested with a simple log likelihood test. The log likelihood *L* for each sample *x* of the tDTSE coming from each model was calculated as:
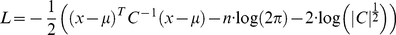
(8)where *n* is the dimensionality of the model, *μ* and *C* are the mean and covariance of the model being tested (either the STSM, DTSM, or sDTSM), log is the natural logarithm, (•)*^T^* represents the transpose of the matrix, and |•| represents the determinant of the matrix. The difference of log likelihood values, *L_DTSM_*-*L_STSM_* and *L_DTSM_*-*L_sDTSM_*, were then calculated to determine the log-likelihood ratios (LLRs). Samples with log likelihood ratios greater than zero were more likely to have been elicited by the data-based model, while samples with log likelihood ratios of zero were equally likely to have been elicited by either model in the test. Prior to performing likelihood tests, all models and test samples were projected into a reduced space to overcome spurious effects due to band-limited stimuli [11], (see supplementary text S1, section ‘Effects of Band-Limiting on Likelihood Analysis’). Due to the large data demands of the multivariate models, we removed ISIs from experiments that had less than 80 samples. In order to avoid biasing due to large outliers we also removed LLR values with absolute values greater than three standard deviations from the mean. This typically amounted to less than 2% of the available samples.

When visualizing the LLR distribution vs the ISI of the respective models, we modeled the observed decay with a sum of exponentials identical to Eq. 2:
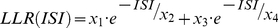
(9)We obtain the parameters of the model as a least-squared fit in a manner identical to the analysis of Eq. 2. This functional relation was selected among several by again using the AIC.

### iSTAC analysis

We employed iSTAC [43] to compactly describe the difference between the DTSM and the sDTSM. iSTAC finds a subspace that maintains as much as possible of the Kullback-Leibler (KL) divergence between two distributions. We briefly summarize the method of Pillow and Simoncelli:

iSTAC assumes that the probability of a stimulus *x* given a certain condition is normal:
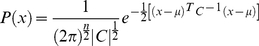
(10)In this probabilistic formulation, the difference between two data sets is characterized by the KL divergence:
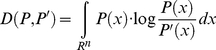
(11)where *P*(*x*) is the base probability against which differences are sought (in our case either the sDTSM or the sDTSM+DTSM, see methods; the STSM was not tested with iSTAC), and *P'*(*x*) is the probability which needs to be discriminated (either the DTSM or the raw stimulus). *D*(*P,P'*) is an information-theoretic quantity charactering the difference between the two distribution in bits (all log values are base 2). Since we were only interested in relative comparison between two distributions, the base probability *P*(*x*) was rescaled to have zero mean and an identity covariance matrix. Therefore, let:
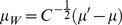
(12)and
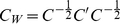
(13)where *μ_W_* and *C_W_* are the DTSM mean and covariance, respectively whitened against the base probability. This allows us to simplify Eq. 11 to:
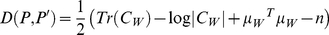
(14)where *Tr*(•) represents the trace of the matrix. We then specify an *m*-dimensional linear subspace defined by an orthonormal basis *B* in which *D(P,P')* satisfies:
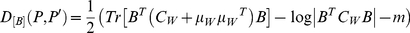
(15)


The most informative subspace is described by the matrix *B* that maximizes Eq. 15. We analyzed a variety of dimensionalities *m*, ranging from single dimensional to the full dimensionality of our models.

## Supporting Information

Figure S1 Measure of the total K-L divergence between data-based and synthetic models for 2, 5, and 8 ms, as a function of subspace dimensionality. Mean ± SD across 10× validation is shown with error bars, which are on the order of the size of the markers for the points. Data presentation is as in Figure 9E, however here the K-L divergence has not been normalized (and so is in units of bits).
(EPS)

Figure S2 Improvement of the synthetic model performance in LLR tests from the data from the 9 neurons shown in Figures 7D and 8B (black markers) by modification along their respective 3-dimensional iSTAC subspaces (cyan markers). Error bars represent ±95% CIs on the mean.
(EPS)

Text S1 Supplementary Methods.
(DOC)
