# Supplementary material for: Temporal Encoding in a Nervous System
Source: PLoS Comput Biol. 2011 May 5;7(5):e1002041. doi: 10.1371/journal.pcbi.1002041 (PMC3088658; doi:10.1371/journal.pcbi.1002041)
Supplement: Text S1 — Supplementary Methods. (DOC) [file pcbi.1002041.s003.doc]

**S1 Supplementary Methods**

*Covariance Structure of Synthetic Stimulus Models*

Since in this work we would like to test for linearity of doublet responses with specific ISIs, the starting point for our analysis and synthetic doublet model construction is the linear stimulus reconstruction method, which speaks of linearity when explaining conditional means of composite events. The benefits of being able to explain doublet (or more complex) models as superpositions of singlets is obvious – there are many more observations with which to constrain singlet models and relatively few with which to build well-behaving full doublet models. Thus, synthesizing a doublet model allows for a more efficient use of data, in addition to elucidating some of the functional properties of the analyzed system.

The original stimulus reconstruction method explicitly defines the two-spike conditional mean to be the sum of single-spike means,

(S1)

where *μ_sD_* is the mean of a synthetic doublet model, and *μ_S_* is the mean of the isolated single spike model. However, reconstruction only defines the noise structure of the spike-triggered signals implicitly, through the least-squared optimization criterion [1]. When adding the condition that the input signal is Gaussian White Noise, this implies that all distributions *(p(s), p(s|r_1_), p(s|r_2_), p(s|r_1_, r_2_))* have covariance of the form *σ*_I_, that is, they have spherical (white) covariance, and that

*C = C_s_ = C_sD_* = *σ*_I_. (S2)

To extend this approach to the more general covariance structures encountered here, we model the linear process as a sum of two random variables, *S_sD_ = S_s(t)_ + S_s(t+ISI)_, S_t_* with mean and covariance *(μ*(*t*)*, C*(*t*)). The means of the two random variables add, so by definition we have, as in (S1). The combined covariance is more involved. In general,

(S3)

where the last term is the co-variation between the random variables *S_1_* and *S_2_*. Here, *t*_1_ and *t*_2_ represent time indices relative to the time of pattern onset, and *ISI* represents the inter-spike interval duration of the desired synthetic model.

Since *S_2_* is a time-shifted version of *S_1_*, there is a lot of co-variation induced by the stimulus variance structure for non-white signals. We would like to avoid determining the covariation from data for the reasons outlined above, and would like to find an approximation of *C_sD_* using only *C_S_* and possibly its shifted versions. If, as a first approximation we decide to disregard the covariation and assume independent conditional distributions (*p*(*s*|*r_1_,r_2_*) = p(*s*|*r_1_*)*p*(*s*|*r_2_*))*,* it implies that

(S4)

In such a case we do not obtain the white noise limit of linear stimulus reconstruction, as when *C_S_* = *σ_I_*, then *C_sD_* = *2C_S_* = *2σ_I_* instead of the required *C* = *C_S_* = *C_sD_* = *σ_I_* from Eq. S2. This implies that we need to assume some form of co-variation. Here we assume that the combined covariance matrix is still a superposition of the two single-spike covariances, and has a form that normalizes the sum of covariances to have the same volume as the single spike models, that is, *cov*(*S_1_,S_2_*) is such that *det*(*C_sD_*) = *det*(*C_S_*). Thus in the model we use

(S5)

where

(S6)

with *n* being the dimensionality of the model. With this normalization, we remove model differences due to gross differences in the size of the modeled space, and only retain differences due to the shapes of the covariance structures. In the limit of *C_S_* = *σ_I_*, the above approximation yields the correct asymptotic, *C_sD_* = *σ_I_*.

Defining *α* as a ratio of determinants is potentially numerically unstable. For actual computations, we use the spectral decomposition of the matrix and express *α* through eigenvalues. From above,

(S7)

The normalization constant *α* is then obtained by exponentiation of the value in Eq. S7. Note that the products and sums in Eq. S7 are over all eigenvalues of the respective covariance matrices. Eq. (S7) was implemented in Matlab® through sum(log(eig(*C*)) for the corresponding covariances. That is equivalent to log(prod(eig(*C*))) as an expression, but is numerically more precise.

*Effects of Band-Limiting on Likelihood Analysis*

The stimulus that we used was band-limited. Consequently, the covariance matrix of a model built from randomly chosen stimulus segments contained significant structure, reflecting correlation between stimulus samples induced by the band limit. Models of stimuli leading up to spikes also included these correlation effects, and these became the dominant features of some models particularly at time lags far from the conditioning spike. In addition, the raw stimulus structure was accounted for more strongly in models based on greater numbers of data segments, and in models conditioned on single spikes versus doublet patterns. At lags greater than 20 ms before the spike in singlet-based models, the off-diagonal covariance structure was nearly identical to that of the unconditioned stimulus.

For sufficiently long ISIs, the effect of modeling unconditioned stimulus structure contributed significantly to the likelihood of generating a given stimulus event under each model. Since models of successively longer ISIs were built from smaller amounts of data (figure 1C of text), the effects of stimulus correlation were not represented equally in all data-based models, and hence they affected the likelihood ratio tests used to compare the synthetic and data-based models. We have no reason to believe that the band-limited structure of our stimulus is directly related to the coding properties of the interneurons, so we viewed this effect as a bias on the likelihood estimations.

Reducing the length of the models greatly reduces this effect, but does not remove it. Additionally, using very short periods of stimulus before a spike might also discard relevant information about the stimulus-response relationship of the neurons. Consequently, we desired a method for reducing the dimensionality of our models which would retain information that distinguished response-conditioned stimuli from raw stimuli, while discarding information that was a result of the structure of the raw stimulus.

We used “information-theoretic spike-triggered average and covariance” (iSTAC) analysis [2] to determine a subspace that retained the stimulus features associated with spiking events, but did not retain features of the raw stimulus structure. After constructing the iSTAC subspace, we projected all STSMs, DTSMs sDTSMs, and test samples into this space where we subsequently calculated the log likelihoods.

In its original use, iSTAC finds a subspace that maintains as much as possible of the Kullback-Leibler (KL) divergence between two distributions. We applied iSTAC in this manner in the main text to compare the DTSM and the sDTSM. However, in order to solve the biasing issue introduced by band-limiting we used iSTAC to discard as much as possible of the common signal structure, by removing the subspace that does not represent major portions of the KL divergence.

When iSTAC was used in this manner, the goal was to remove dimensions in which the conditional and raw stimuli were not discriminable, rather than find and interpret highly discriminable directions. In this case, the dimensionality of the subspace (see methods, [3]) *m* was reduced to the subspace that contained 90% of the retained KL cost. Typically that reduced the dimensionality of the models to *m≈n/4*. The base probability *P*(*x*) was the unconditioned signal probability (GWN, normal with zero mean and covariance characterizing the band-limited nature of the signal). The discriminating probability *P’*(*x*) was selected so as to retain as much detail as possible about the distribution of both real and synthetic doublets. To obtain parameters in an unbiased manner, for each analyzed cell we created a dataset that contained an equal number of doublet-conditioned stimuli from both the sampled data and the sDTSMs, for all analyzed doublets. The number of samples per doublet was restricted to match the dataset with fewest samples (typically, the one with longest ISI). The parameters of *P’*(*x*) were then estimated from that dataset, and used to build the iSTAC subspace for dimensionality reduction.

**References**

1. Rieke F, Warland D, Bialek W, de Ruyter van Steveninck RR (1997) Spikes: exploring the neural code. Cambridge, Mass. ; London: MIT Press.

2. Pillow J, W., Simoncelli E, P. (2006) Dimensionality reduction in neural models: An information-theoretic generalization of spike-triggered average and covariance analysis. Journal of Vision J Vis 6: 414-428.

3. Dimitrov A, Cummins G, Baker A, Aldworth Z (2011) Characterizing the fine structure of a neural sensory code through information distortion. Journal of Computational Neuroscience 30: 163-179.
